# Hematogones in CD34+ leukapheresis units: Is it important to quantify and report them?

**DOI:** 10.1016/j.htct.2026.106479

**Published:** 2026-05-23

**Authors:** Rebeca Brasil Albuquerque, Hélio Lopes da Silva, Fernando Barroso Duarte, Daniel Mazza Matos

**Affiliations:** a Flow Cytometry Section, Cell Processing Center (CPC), Center of Hematology and Hemotherapy of Ceará (HEMOCE), Fortaleza, Ceará, Brazil; b Universidade de Fortaleza (UNIFOR), Fortaleza, Ceará, Brazil

Dear Sir,

The quantification of CD34^+^ hematopoietic stem cells (HSCs) plays a critical role in the feasibility of hematopoietic stem cell transplantation (HSCT). In general, most centers collect a minimum of 2.0 and 4.0 × 10^6^ CD34^+^ HSCs per kilogram of recipient body weight for autologous and allogeneic HSCT, respectively. In this scenario, normal B-cell lymphoid precursors, also known as hematogones, are sometimes identified by flow cytometry in mobilized leukapheresis units. As hematogones are characterized by the expression of the CD34 antigen, their presence in apheresis products scheduled for infusion in patients undergoing HSCT raises the question of whether, in fact, a sufficient number of bona fide CD34^+^ HSCs has been collected [1].

From January 2020 to January 2024, the Flow Cytometry Section of the Center of Hematology and Hemotherapy of Ceará (CPC–HEMOCE) received a total of 501 leukapheresis units. These samples were systematically evaluated to identify cases in which the detection of hematogones was essential to avoid proceeding with HSCT using a suboptimal dose of CD34^+^ HSCs. All specimens included in this study were part of the routine activities of the CPC and were handled under the auspices of the HEMOCE. Anonymity and confidentiality of the information were maintained.

The enumeration of CD34^+^ HSCs was performed in accordance with the International Society of Hematotherapy and Graft Engineering (ISHAGE) guidelines using three flow cytometers: BD FACSVia (BD Biosciences), BD FACSCanto II (BD Biosciences), and DxFLEX (Beckman Coulter). On the BD FACSVia and BD FACSCanto II, CD34^+^ HSC quantification was carried out using the single-platform BD Stem Cell Enumeration Kit (BD Biosciences) [2], prepared according to the manufacturer’s instructions and following the ISHAGE sequential gating strategy. Briefly, 20 µL of a pooled monoclonal antibody reagent containing anti-CD45-FITC (clone 2D1) and anti-CD34-PE (clone 8G12), along with 20 µL of 7-aminoactinomycin D (7-AAD) for viability assessment, were added to the bottom of a BD Trucount tube. Next, 100 µL of the sample was pipetted onto the side of the tube, just above the bead retainer. Erythrocytes were lysed by adding 2 mL of 1 × ammonium chloride lysing solution. The sample was incubated for 10 min at 20–25 °C, protected from light. For CD34^+^ HSC enumeration on the DxFLEX, the single-platform Stem-Kit (Beckman Coulter) was prepared according to the manufacturer’s recommendations. Briefly, 20 µL of a pooled monoclonal antibody reagent containing anti-CD45-FITC (clone J33) and anti-CD34-PE (clone 581), together with 20 µL of 7-AAD, were pipetted into the bottom of the tube. Erythrocytes were lysed by adding 2 mL of the Stem-Kit lysing solution, followed by incubation for 10 min at 20–25 °C, protected from light. Subsequently, 100 µL of Stem-Count Fluorospheres was added to the tube. Data acquisition and analysis were performed using an ISHAGE-based gating template, with a minimum of 100 CD34^+^ events and at least 150,000 CD45^+^ events being acquired per sample.

To detect hematogones, an additional gate was created to include events characterized by CD34 positivity, low-intensity CD45 expression, very low forward scatter (FSC), and very low side scatter (SSC). This profile corresponds to the typical immunophenotype of hematogones (Figure 1).

**Figure 1:**
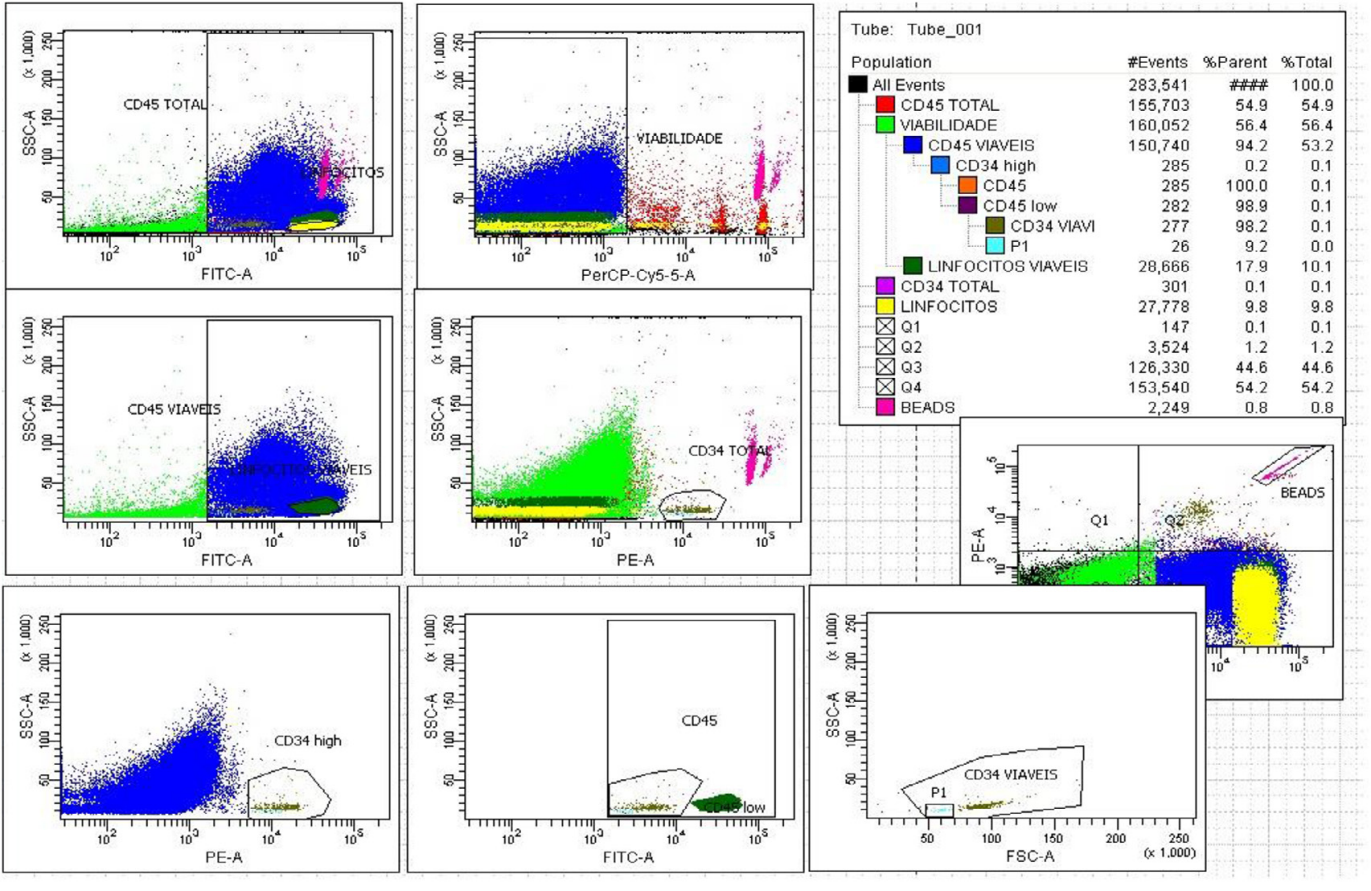
Enumeration of CD34^+^ hematopoietic stem cells (HSCs) and hematogones using a single-platform ISHAGE-based protocol.

Data were collected on a BD FACS Canto II. An extra-gate (P1) was designed to include hematogones. Notice the very low forward scatter (FSC) and side scatter (SSC) of hematogones (light blue) when compared with CD34^+^ HSCs (green). Presence of 9.2 % of hematogones

Hematogones were detected in 61 (12.1 %) of the 501 leukapheresis units analyzed. In three cases, the identification and quantification of hematogones were of paramount importance to avoid the infusion of a suboptimal dose of CD34^+^ HSCs (Table 1).

**Table 1:** Profiles of three autologous hematopoietic stem cell transplantation patients with borderline CD34^+^ counts due to hematogones.

Case	Age / gender	Mobilization	Diagnosis	1 °Collection (CD34+ cells/kg)	Hematogones (%)	1 °Collection (CD34^+^ cells/kg) *	2 °Collection (CD34^+^ cells/kg) *	Total (CD34^+^ cells/kg) *
1	65 / female	G-CSF (1.200 mg/d)	Multiple myeloma	2.0 × 10⁶	9.2	1.8 × 10⁶	0.8 × 10⁶	2.6 × 10⁶
2	62 / female	G-CSF (1.500 mg/d)	Multiple myeloma	2.0 × 10⁶	15.8	1.6 × 10⁶	0.9 × 10⁶	2.5 × 10⁶
3	23 / male	G-CSF (1.200 mg/d)	Hodgkin lymphoma	2.6 × 10⁶	21.8	2.0 × 10⁶	0.6 × 10⁶	2.6 × 10⁶

⁎ CD34^+^ cell counts were adjusted to exclude hematogones.

Case 1 was a 65-year-old female patient scheduled to undergo autologous HSCT for multiple myeloma. The first leukapheresis collection yielded 2.0 × 10⁶ CD34^+^ HSC/kg, of which 9.2 % were identified as hematogones. After correction for the hematogone percentage, the effective CD34^+^ HSCs dose decreased to 1.8 × 10⁶ CD34^+^ HSC/kg. On this basis, the transplant physician deemed it necessary to perform a second leukapheresis procedure. The second collection yielded 0.8 × 10⁶ CD34^+^ HSC/kg, resulting in a final infused dose of 2.6 × 10⁶ CD34^+^ HSC/kg.

Case 2 was a 62-year-old female patient also undergoing autologous HSCT for multiple myeloma. The first leukapheresis yielded 2.0 × 10⁶ CD34^+^ HSC/kg, of which 15.8 % were identified as hematogones. After correction for the hematogone percentage, the effective CD34^+^ HSCs dose decreased to 1.6 × 10⁶ CD34^+^ HSC/kg. The second collection yielded 0.9 × 10⁶ CD34^+^ HSC/kg, resulting in a final infused dose of 2.5 × 10⁶ CD34^+^ HSC/kg.

Case 3 was a 23-year-old male patient scheduled for autologous HSCT for Hodgkin lymphoma. The first leukapheresis collection yielded 2.6 × 10⁶ CD34^+^ HSC/kg, a dose considered acceptable for autologous HSCT. Hematogones accounted for 21.8 % of CD34^+^ events. After correction for the hematogone percentage, the effective CD34^+^ HSCs dose decreased to 2.0 × 10⁶ CD34^+^ HSC/kg, representing the minimum value for proceeding with autologous HSCT. Consequently, the transplant physician requested an additional leukapheresis procedure that yielded 0.6 × 10⁶ CD34^+^ HSC/kg, resulting in a final infused dose of 2.6 × 10⁶ CD34^+^ HSC/kg.

To date, there is no definitive clinical evidence in the literature regarding the optimal management of leukapheresis units containing hematogones. Studies by Nadeem et al. [3] and Ondrejka et al. [4] evaluated the relationship between the presence of hematogones in leukapheresis units and neutrophil and platelet engraftment times. However, neither study examined whether reduced CD34+ cell doses, caused by the presence of hematogones, led to delayed or impaired engraftment. In light of this uncertainty, we consider it essential to provide transplant physicians with corrected CD34^+^ HSCs counts, adjusted for the proportion of hematogones, in order to highlight potentially suboptimal graft doses.

Thus, until definitive evidence is available regarding the relationship between hematogones and engraftment kinetics, we strongly recommend that laboratories involved in CD34^+^ HSCs enumeration (a) routinely assess the presence of hematogones in all leukapheresis samples and (b) call attention to their presence in laboratory reports, particularly when hematogones substantially reduce the effective CD34^+^ HSCs dose to a level considered inadequate for HSCT. This practice would alert transplant physicians to the actual number of bona fide CD34^+^ HSCs in leukapheresis units scheduled for infusion.

## Data availability

The data that support the findings of this study are available from the corresponding author upon reasonable request.

## Conflicts of interest

The authors declare no conflict of interest.
